# Peculiaridade dos Pacientes com Arritmias Hereditárias na Pandemia pela COVID-19

**DOI:** 10.36660/abc.20200391

**Published:** 2021-08-09

**Authors:** Luciana Sacilotto, Natalia Quintella Sangiorgi Olivetti, Cristiano Faria Pisani, Tan Chen Wu, Ludhmila Abrahão Hajjar, Sissy Lara de Melo, Sávia Christina Pereira Bueno, Esteban Wisnivesky Rocca Rivarola, Muhieddine Omar Chokr, Carina Abigail Hardy, Denise Tessariol Hachul, Francisco Carlos da Costa Darrieux, Mauricio Ibrahim Scanavacca

**Affiliations:** 1 Universidade Federal dos Vales do Jequitinhonha e Mucuri Faculdade de Medicina da Universidade de Instituto do Coração do Hospital das Clínicas da São PauloSP Brasil Instituto do Coração do Hospital das Clínicas da Faculdade de Medicina da Universidade de São Paulo, São Paulo, SP - Brasil.

**Keywords:** COVID-19, Cardiomiopatia Arritmogênica do Ventrículo Direito, Síndrome de Brugada, Síndrome do QT Longo, Taquicardia Ventricular Polimórfica Catecolaminérgica

## Abstract

Desde dezembro de 2019, observamos o rápido avanço da síndrome respiratória aguda grave causada pelo coronavírus 2019 (SARS-CoV-2). O impacto da evolução clínica de uma infecção respiratória é pouco conhecido em pacientes portadores de arritmias hereditárias, devido à baixa prevalência dessas doenças. Os pacientes que apresentam quadros infecciosos podem exacerbar arritmias primárias ocultas ou bem controladas, por diversos fatores, tais como febre, distúrbios eletrolíticos, interações medicamentosas, estresse adrenérgico e, eventualmente, o próprio dano miocárdico do paciente séptico. O objetivo desta revisão é destacar os principais desafios que podemos encontrar durante a pandemia pela Covid 19, especificamente nos pacientes com arritmias hereditárias, com destaque para a síndrome do QT longo congênito (SQTL), a síndrome de Brugada (SBr), a taquicardia ventricular polimórfica catecolaminérgica (TVPC) e a cardiomiopatia arritmogênica do ventrículo direito.

Desde dezembro de 2019, observamos o rápido avanço da síndrome respiratória aguda grave causada pelo novo coronavírus (SARS-CoV-2), que registrou os primeiros casos em Wuhan, na China, e atingiu o Brasil recentemente. Estudos retrospectivos demonstraram que a idade avançada foi um preditor de mortalidade independente na Covid-19. Os demais fatores de risco, com impacto na mortalidade, foram hipertensão arterial sistêmica, doença pulmonar obstrutiva crônica, imunossupressão, diabetes melito tipo 2, obesidade e cardiopatia severa (insuficiência cardíaca, doença coronária ou miocardiopatias).^1^^,^^2^

De forma geral, as complicações por arritmias em pacientes com pneumonia são relativamente comuns, destacando-se a fibrilação atrial.^3^^,^^4^ A parada cardíaca ocorre em cerca de 3% dos pacientes internados,^5^ porém menos de 20% dos eventos hospitalares são descritos como ritmo passível de reversão elétrica (cardioversão ou desfibrilação), ou seja, taquicardia/fibrilação ventricular (TV/FV).^6^ Nesses pacientes, o principal mecanismo arritmogênico é o dano miocárdico por isquemia ou inflamação.^4^

O impacto da evolução clínica da sepse, ou particularmente das infecções respiratórias, é pouco conhecido em pacientes portadores de arritmias hereditárias, devido à baixa prevalência dessas doenças.^7^ Além disso, em sua maioria, as arritmias hereditárias apresentam, em geral, penetrância incompleta e idade dependente,^8^ sendo mais expressas em pacientes jovens que, por sua vez, apresentam risco menor de desenvolver quadros infecciosos graves.

Os pacientes que apresentam quadros infecciosos mais graves podem exacerbar arritmias ocultas ou bem controladas, por diversos fatores, tais como febre, distúrbios eletrolíticos, interações medicamentosas, estresse adrenérgico e, eventualmente, o próprio dano miocárdico do paciente séptico. Todos esses fatores podem alterar o equilíbrio dos canais iônicos, tornando os pacientes com arritmias hereditárias potencialmente mais vulneráveis.

Os eventos fatais em pacientes com arritmias hereditárias podem ser desencadeados por estresse físico e emocional. Os impactos psicossociais da pandemia relacionados a depressão, estresse, ansiedade e síndrome do pânico, agravados pelo isolamento social, enfrentamento do medo e do luto, podem predispor a maior ocorrência de arritmias.^9^ A eventual necessidade de suspensão temporária das medicações (betabloqueadores e antiarrítmicos) em pacientes com instabilidade hemodinâmica, uso de fármacos vasopressores com efeito catecolaminérgico e distúrbios hidroeletrolíticos pode estar associada a maior risco de eventos potencialmente fatais. Portanto, o período da pandemia, por si só, já alerta para a necessidade de garantir vigilância e orientações direcionadas para esses pacientes, que, por serem portadores de doenças raras, não serão bem representadas em estudos clínicos. Em caso de infecção pelo SARS-COV2, não há dados epidemiológicos suficientes nessa população para categorizar os pacientes em grupo de risco, gerando insegurança para o médico e para o paciente. Na Tabela 1, elencamos os cuidados gerais intra e extra-hospitalares que podem ser adotados nos pacientes com arritmias genéticas previamente conhecidas.

**Tabela 1:** Cuidados sugeridos durante a pandemia de Covid-19 em pacientes com arritmias hereditárias

**Cuidados ambulatoriais**
• Evitar excesso de notícias alarmistas e estresse emocional
• Manter os hábitos de higiene e isolamento social
• Avaliar o risco do isolamento social
• Manter ou otimizar betabloqueadores e antiarrítmicos, sempre que possível
• Vigilância diária na aderência terapêutica e sinais de alarme para arritmias (síncope, pré-síncope e palpitações)
**Cuidados ambulatoriais e hospitalares**
• Manter eutermia – a febre deve ser tratada precocemente, principalmente em portadores de SQTL2 e SBr
• Em portadores de SQTL, desencorajamos o uso de azitromicina, hidroxicloroquina/cloroquina
• Evitar a associação de hidroxicloroquina com amiodarona ou sotalol pelo risco de TdP, comuns em pacientes com CAVD
• Acessar o site www.credibledrugs.org para consulta de medicações que prolongam o intervalo QT
• Acessar o site www.online.epocrates.com para consulta de interações medicamentosas
• Atenção ao protocolo de controle de QT de cada instituição

SQTL: síndrome do QT longo; SBr: síndrome de Brugada; CAVD: cardiomiopatia arritmogênica do ventrículo direito; TdP: torsades de pointes

Assim, é importante revisar os principais desafios que podemos encontrar durante a pandemia da Covid 19,^7^ especificamente nessa subpopulação, com destaque para a síndrome do QT longo congênito (SQTL), a síndrome de Brugada (SBr), a taquicardia ventricular polimórfica catecolaminérgica (TVPC) e a cardiomiopatia arritmogênica do ventrículo direito (CAVD).

## Síndrome do QT longo congênito

### Aspectos gerais

A SQTL ocorre em 1 em 2.000 pessoas, é caracterizada pelo prolongamento do intervalo QT no eletrocardiograma (ECG) de repouso de 12 derivações e uma propensão para síncope ou convulsões por *torsades de pointes* (TdP) e morte súbita cardíaca (MSC).^10^

Está associada a defeitos que acentuem as correntes despolarizantes de sódio e cálcio (INa e ICa L, respectivamente) ou atenuem correntes repolarizantes de potássio (IKs, IKr e IK1), levando a um prolongamento no potencial de ação cardíaco, refletindo no intervalo QT prolongado.^11^ Há o reconhecimento de peculiaridades clínicas, que permitem classificar a SQTL em subtipos, principalmente SQTL 1 (canal IKs, gene *KCNQ1*), 2 (canais hERG ou IKr, gene *KCNH2*) e 3 (canal INa, gene *SCN5A*).

**Figura 1:**
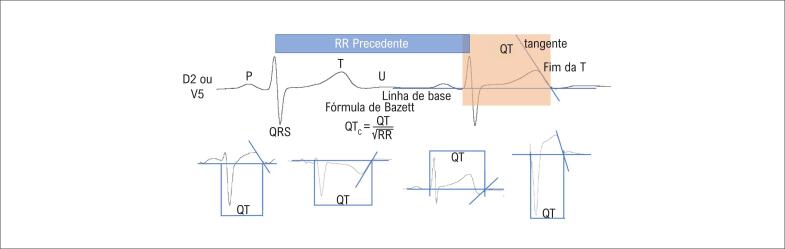
Medida do intervalo QT em paciente com síndrome do QT longo.

A medida do intervalo QT deve ser preferencialmente realizada nas derivações DII ou V5 (Figura 1), corrigida pelo intervalo RR precedente, utilizando a fórmula de Bazzet, preferencialmente em frequência cardíaca entre 60 a 80bpm.^12^ As diretrizes atuais definem um valor prolongado de QTc quando acima e 450 ms em homens e 460 ms em mulheres. No entanto, 5% a 10% dos indivíduos saudáveis têm um QTc >460ms, sendo necessários outros dados clínicos para compor o diagnóstico de SQTL. Nesses casos, recomenda-se a utilização do escore de Schwartz.^13^^,^^14^ Apenas quando os valores de QTc são maiores que 480ms, na ausência de causas secundárias, há maior especificidade para a SQTL.^14^ Por outro lado, cerca de 30% dos pacientes têm a forma oculta da SQTL, representada por alteração genética e intervalo QT normal, sendo o histórico familiar ou o teste genético relevantes para suspeita clínica nesses pacientes.

Fonte: Fonte: acervo InCor-HC-FMUSP.

A conduta padrão em paciente com SQTL é o bloqueio beta-adrenérgico, com uso de nadolol/propranolol, além de evitar medicações que prolonguem o intervalo QT. A indicação de denervação simpática cardíaca esquerda e do cardiodesfibrilador implantável (CDI) permanece reservada nos casos de maior risco de eventos arrítmicos potencialmente fatais.^14^

### Cuidados na pandemia pela Covid-19

Os canais iônicos cardíacos são modulados pelo sistema nervoso autônomo, pois o tempo de repolarização cardíaca é constantemente ajustado pela frequência cardíaca. Em situações de estresse adrenérgico, temos aumento da frequência cardíaca, com fosforilação dos canais de potássio e aumento da sua velocidade de abertura em condições normais. Em situações de defeitos proteicos geneticamente determinados, esse ajuste é prejudicado, lentificando a negativação do potencial de membrana, o que permite a entrada desequilibrada de cálcio e ocorrência de pós-potenciais precoces. Enquanto a frequência cardíaca permanece elevada, pode haver alguma inibição desses potenciais, entretanto quando a situação metabólica leva a bradicardia ou irregularidade do intervalo RR, observamos um aumento da dispersão da repolarização e maior ocorrência de pós-potenciais de fase 3. Dependendo do limiar de excitabilidade da célula, os pós-potenciais geram extrassístoles ventriculares, TdP/FV.^11^

A suspensão ou redução de betabloqueadores usados cronicamente no tratamento da SQTL pode agravar a ocorrência de arritmias potencialmente fatais. Portanto, as medicações devem ser rigorosamente mantidas em pacientes em tratamento domiciliar e, no doente crítico, a decisão deve ser pautada pela estabilidade hemodinâmica.

O equilíbrio das correntes iônicas depende do nível celular desses íons, que pode ser dinâmico em doentes críticos. A hipocalemia, a hipocalcemia e a hipomagnesemia provocam pós-potenciais precoces, aumentam a dispersão da repolarização e levam a TdP com mais facilidade em portadores de SQTL. A manutenção do potássio entre 4,5 a 5mg/dL pode ser uma estratégia protetora.^7^

A sepse ou o choque séptico são estados adrenérgicos por dor/desconfortos, além do próprio estado inflamatório. Os portadores de SQTL 1 e SQTL 2 são os que apresentam arritmias desencadeadas principalmente por estresse adrenérgico e, portanto, mais vulneráveis nessas situações críticas. A presença da febre pode influenciar as propriedades biofísicas dos canais sensíveis à temperatura, particularmente os canais hERG, acometidos na SQTL2.^15^ Por outro lado, a hipotermia também está associada ao prolongamento do intervalo QT, porém com baixo risco de indução de TdP.^16^

As lesões miocárdicas inflamatórias alteram o potencial de membrana celular, gerando dispersão de repolarização e suscetibilidade às arritmias ventriculares.^17^ Além disso, citocinas e autoanticorpos podem se ligar aos canais iônicos cardíacos levando à canalopatia induzida pela inflamação, com maior gravidade presumida em pacientes com SQTL.^18^

Os fármacos que bloqueiam os canais de potássio (hERG) podem prolongar ainda mais a repolarização cardíaca, aumentando o risco de arritmias fatais. A indicação dessas medicações deve ser rigorosamente ponderada, principalmente em regime extra-hospitalar, pela falta de monitoramento contínuo. Além disso, os medicamentos que levam à inibição do citocromo P450 3A4 (CYP3A4) podem aumentar ainda mais o nível plasmático de fármacos que prolongam o intervalo QT.^7^

A lista de medicações está disponível em www.crediblemeds.org e inclui a cloroquina/hidroxicloroquina e a azitromicina. No doente crítico, outras medicações de risco são frequentemente administradas, tais como antieméticos (ondasentrona e metoclopramida),^19^ antipsicóticos (haloperidol), fármacos vasoativos (noradrenalina, dobutamina), analgésicos (tramadol) e sedativos (etomidato, propofol).^20^

A cloroquina e a hidroxicloroquina, associadas ou não à azitromicina, apresentam uso controverso em pacientes com Covid-19, sendo sua eficácia demonstrada *in-vitro*,^21^ porém ainda sem estudos clínicos comprobatórios. Em recente publicação, Mazzanti et al. sugerem que uma dose cumulativa de hidroxicloroquina de 2g em 5 dias, conforme adotada em 30% de todos os estudos em andamento com hidroxicloroquina (www.clinicaltrials.gov), leva a um prolongamento modesto do intervalo QTc em pacientes com QTc basal normal (média de aumento de 20ms) e sem aumento do risco de complicações arrítmicas ameaçadoras à vida.^22^ Em outra série de pacientes com lúpus eritematoso sistêmico, com intervalo QTc médio de 443±25,3ms (373 – 518ms), houve um prolongamento do intervalo QTc em 14,2% dos pacientes em uso de cloroquina.^23^ Considerando que os pacientes com SQTL já apresentam maior suscetibilidade a pró-arritmias tipo *TdP*, o uso da cloroquina ou hidroxicloroquina, principalmente quando associado à azitromicina, deve ser desencorajado em pacientes com SQTL.^12^

A polifarmácia é um item que exige precaução multidisciplinar, por parte de médicos e farmacêuticos; em pacientes com SQTL, isso se torna uma preocupação ainda mais relevante pelo risco iminente de morte súbita se expostos a tais fármacos. Especificamente nesses pacientes, devemos discutir pontualmente riscos e benefícios de cada medicação.

Em caso de ocorrência de TdP com degeneração para FV, há necessidade de desfibrilação e medidas de reanimação cardiopulmonar. O TdP costuma ter uma apresentação autolimitada com resolução espontânea; entretanto, o mais desafiador é a prevenção da recorrência do *TdP*. As medidas urgenciais incluem minimizar medicações pró-arrítmicas e suprimir os fatores que geram pós-potenciais precoces.^24^

A principal medida para a supressão dos pós-potenciais precoces na SQTL é evitar bradicardia e “pausa-dependência”. Em pacientes com SQTL adquirida, é possível tentar medidas farmacológicas, como isoprenalina endovenosa; por outro lado, em pacientes com SQTL congênita, o *overdriving* deve ser realizado apenas por meio de estímulo atrial ou ventricular, com marca-passo provisório (transcutâneo ou transvenoso). A administração de 2g sulfato de magnésio, seguida de infusão contínua (3 a 10mg/min) é terapia coadjuvante em ambas as circunstâncias, com objetivo de reduzir a amplitude da oscilação do potencial de membrana na fase 3 do potencial de ação. Em caso de refratariedade, a sedação para cessar o estímulo adrenérgico pode ser necessária.^25^

## Síndrome de Brugada

### Aspectos gerais

**Figura 2:**
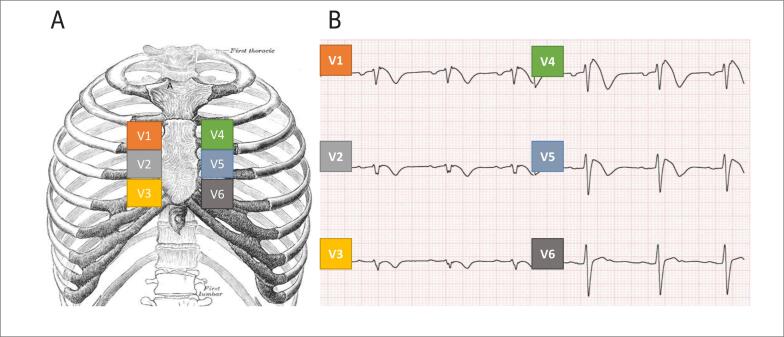
Posicionamento dos eletrodos precordiais na síndrome de Brugada. A. Posicionamento das derivações precordiais para se obter maior sensibilidade na detecção do padrão típico de Brugada, demonstrado no eletrocardiograma (ECG) em B, com típico supradesnivelamento do segmento ST. Paciente de 26 anos de idade, assintomática, com antecedente familiar de morte súbita precoce.

A SBr acomete cerca de 1 em 5.000 pessoas, com predomínio no sexo masculino. O diagnóstico da SBr é definido pelo ECG, na presença de supradesnivelamento do segmento ST de 2mm, em pelo menos uma derivação precordial direita (V1-V2), em posição padrão (quarto espaço intercostal [EIC]) ou em EIC superiores (segundo ou terceiro EIC) (Figura 2), seguido de onda T negativa (padrão tipo 1). O principal desafio no diagnóstico e na classificação é que esse padrão eletrocardiográfico é dinâmico na maioria dos pacientes;^14^ portanto, pode ser documentado espontaneamente ou necessitar de teste provocativo, sob uso de fármacos específicos (p. ex., ajmalina).

Fonte: acervo InCor-HC-FMUSP.

Na ausência de causas secundárias, a presença do padrão eletrocardiográfico espontâneo de SBr tipo 1 é suficiente para o diagnóstico. Nos casos de SBr induzida por febre ou por testes provocativos, haveria a necessidade de adicionar dados clínicos pessoais e familiares para definir o diagnóstico com precisão. Recentemente, foi proposto um sistema de pontuação na SBr (escore de Shanghai), que pode ser utilizado como ferramenta diagnóstica.^26^

As causas secundárias, chamadas de fenocópias de SBr, incluem uso de fármacos que induzem ao supradesnivelamento do segmento ST (p. ex., tricíclicos), distúrbios eletrolíticos, isquemia miocárdica e outras formas de distorção do segmento ST (presença de *pectus excavatum*) (Tabela 2).^27^

**Tabela 2:** Fenocópias de síndrome de Brugada

**Condições metabólicas**
Crise adrenal, acidose metabólica, hiperglicemia
**Distúrbios eletrolíticos**
Hipercalemia, hipocalemia e hiponatremia
**Compressão mecânica na via de saída do ventrículo direito**
Tumores, *pectus excavatum*
**Outras**
Isquemia miocárdica e embolia pulmonar
Pericardite e miocardite
Medicações e intoxicações exógenas

Fonte: www.brugadadrugs.org^50^

A genética da Sbr é mais complexa do que as outras síndromes elétricas primárias. O SCN5A, primeiro gene identificado, ainda permanece como gene causal, mas todos os demais 20 genes citados na literatura recente carecem de correlação genótipo-fenótipo.^28^

O tratamento para os pacientes com SBr envolve evitar situações que facilitem a ocorrência das arritmias potencialmente fatais (TV/FV), como febre, uso de drogas ilícitas, libação alcoólica, refeições copiosas ou medicações que aumentem o bloqueio dos canais de sódio. A quinidina, importante bloqueador dos canais Ito, aparenta ser segura e reduz eventos arrítmicos no seguimento clínico de pacientes com alto risco.

A indicação CDI é reservada para pacientes que ou apresentaram TV sustentada espontânea documentada, parada cardiorrespiratória (PCR) recuperada ou, na prevenção primária, naqueles que apresentam maior risco de eventos arrítmicos, como na presença da síncope. O estudo eletrofisiológico (EEF) pode ser usado para estratificação dos pacientes assintomáticos, com resultados controversos.^14^ A ablação por radiofrequência do substrato da Sbr emergiu como tratamento coadjuvante para arritmias ventriculares recorrentes e está sendo estudada em pacientes sem eventos arrítmicos prévios.^29^

### Cuidados na pandemia pela Covid-19

O primeiro aspecto em pacientes com SBr é a precisão diagnóstica, por se tratar de uma alteração eletrocardiográfica inespecífica, com vieses de interpretação. A lista de doenças que podem mimetizar a alteração eletrocardiográfica da SBr, chamadas de fenocópias, deve ser cuidadosamente analisada, para adequada orientação (ver Tabela 2). Podemos notar que várias fenocópias podem ocorrer no contexto infeccioso, como as miocardites, os distúrbios eletrolíticos, o tromboembolismo pulmonar e o infarto do miocárdico.

**Figura 3:**
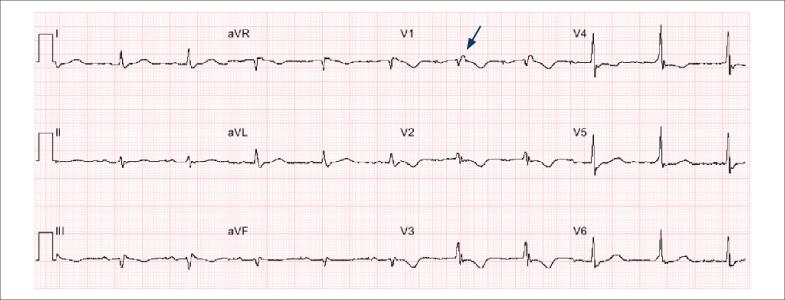
Eletrocardiograma de paciente com cardiomiopatia arritmogênica de ventrículo direito. Paciente de 45 anos de idade, recuperado de parada cardíaca. Observa-se a presença de onda épsilon (seta) e onda T negativa de V1 a V3, ambos critérios maiores, além de baixa voltagem em plano frontal, denotando grave acometimento ventricular.

A posição de eletrodos, descrita na Figura 3, aumenta a sensibilidade diagnóstica de SBr e pode ser preferida em relação à posição de eletrodos-padrão para pacientes com diagnóstico suspeito ou confirmado, podendo ser realizado em pacientes com Covid-19 que apresentem parada cardíaca em TV/FV durante internação em unidade de terapia intensiva (UTI), especialmente quando associada a quadros febris. Por outro lado, para análise de outras patologias, comuns no curso da infecção, como miocardite e infarto, a escolha dos eletrodos em posição superiores deve estar bem identificada no ECG, para não induzir a erros de interpretação com relação à progressão da onda R e amplitude dos complexos. A medida do intervalo QT pode ser realizada na derivação D2 no ECG com derivações superiores, visto que as derivações periféricas são mantidas em posição padrão.

Fonte: acervo InCor-HC-FMUSP.

As principais medidas para manejo dos pacientes com SBr em UTI são prevenir a febre e evitar uso de medicações que acentuem o defeito do canal. Vários são os exemplos de medicações que aumentam risco de morte súbita nos portadores de SBr, como fármacos de suporte no paciente internado (difenidramina), no tratamento de arritmias (amiodarona e propafenona), agentes anestésicos (propofol) e analgésicos (tramadol).

A importância da febre nos pacientes com SBr está bem estabelecida. Em geral, há aumento do intervalo PR, da duração do QRS e aumento do ponto J. A febre aumenta, inclusive, o risco de arritmias em maiores de 70 anos, quando o risco da doença já está reduzido.^30^ Pacientes considerados de alto risco, com temperatura acima de 38,5ºC, apesar do uso de antipiréticos, devem procurar atendimento médico.^7^

A SBr foi a primeira arritmia genética a ganhar destaque em publicações durante a pandemia. Foi relatado o caso de um paciente que desmascarou o padrão eletrocardiográfico durante um estado febril. Por se tratar de um paciente jovem, sem critérios de internação hospitalar, os autores optaram por dar alta com *LifeVest*, indisponível no Brasil.^31^

A hidroxicloroquina e a azitromicina podem ser indicadas, a depender da avaliação de riscos e benefícios. Sugerimos dosar eletrólitos sanguíneos e considerar monitoramento durante o tratamento. O prolongamento do intervalo QT e a dispersão da repolarização com hidroxicloroquina e azitromicina poderiam aumentar o risco de arritmia em pacientes com SBr, mesmo sem relação direta dessas medicações com os canais despolarizantes.^23^^,^^32^

O tratamento da tempestade elétrica na Sbr tem como objetivo aumentar a corrente de cálcio (despolarizante), a fim de se obter normalização do supradesnivelamento do segmento ST e reduzir a reentrada de fase 2. O isoproterenol, como agonistas beta-adrenérgicos, pode ser eficaz, preferencialmente em associação com quinidina (indisponível no Brasil). Os inibidores da fosfodiesterase III, como o cilostazol oral ou o milrinone endovenoso, apresentaram redução de arritmogenicidade em modelos experimentais de Sbr, mas seus efeitos em humanos ainda estão em andamento.^33^

## Cardiomiopatia arritmogênica do ventrículo direito e/ou esquerdo

### Aspectos gerais

A cardiomiopatia arritmogênica do ventrículo direito e/ou esquerdo, também conhecida como displasia ou cardiomiopatia arritmogênica do ventrículo direito (CAVD/DAVD), tem uma prevalência média de 1:5000 na população geral.^34^ É uma doença heterogênea, com diversas apresentações clínicas que pode ter como primeira manifestação a morte súbita cardíaca, que ocorre com maior frequência durante exercício físico. Não há um exame padrão-ouro no diagnóstico de CAVD, e o escore consagrado engloba história clínica, alterações eletrocardiográficas da despolarização e repolarização (Figura 3), exames de imagem, avaliação anatomopatológica e molecular.^35^

Atualmente, sabe-se que a CAVD é uma condição de herança geneticamente determinada, autossômica dominante na maioria dos casos, podendo ter formas raras que são de herança autossômica recessiva, como na doença de Naxos^36^ ou na síndrome de Carvajal.^37^ A penetrância da CAVD é incompleta, com acometimento familiar em até 50% dos casos, podendo estar subestimada pela expressividade variável da doença.

A apresentação clínica da CAVD ocorre frequentemente entre a segunda e a quarta década de vida.^38^^,^^39^ As manifestações mais comuns são palpitações, síncope e parada cardiorrespiratória, podendo progredir para insuficiência cardíaca congestiva.

As mutações relacionadas à CAVD acometem tipicamente genes que codificam proteínas desmossomais, estruturas importantes na adesão celular do cardiomiócito que têm um papel-chave na sua fisiopatologia. Os desmossomos, além de serem estruturas especializadas em conexão celular, são importantes mediadores que atuam na transdução dos sinais intracelulares e intercelulares.^40^ A perda da complexa função desmossomal provoca ruptura da junção intercelular, desprendimento de miócitos e morte celular. A substituição do cardiomiócito por fibrose e gordura contribui para o desenvolvimento de área de condução lenta, que gera substrato anatômico cicatricial para macrorreentrada e arritmias ventriculares. A fibrose progride do epicárdio para o endocárdio e envolve principalmente a parede livre do VD, resultando em afilamento e dilatação aneurismática.^41^

O tratamento deve ser direcionado à manifestação clínica da CAVD. Não há evidência de que os antiarrítmicos previnam a morte súbita, sendo o CDI indicado em pacientes de alto risco (recuperados de PCR e TV espontânea documentada). Os betabloqueadores são considerados primeira linha no tratamento das arritmias atriais, extrassístoles ventriculares, TV não sustentada, além de importante coadjuvante no controle de choques apropriados ou inapropriados (principalmente por arritmias atriais) pelo CDI. O sotalol, a amiodarona e a ablação por radiofrequência podem ser alternativas terapêuticas, quando os betabloqueadores são ineficazes ou mal tolerados.^42^

### Cuidados na pandemia pela Covid-19

As arritmias ventriculares em pacientes com CAVD são frequentemente desencadeadas por estresse físico e emocional, tendo um componente adrenérgico-dependente importante. Dessa forma, o aumento da liberação adrenérgica relacionado à reposta compensatória da síndrome inflamatória que acompanha o quadro infeccioso pode ser indutor das arritmias ventriculares. Deve-se manter o betabloqueador nesses pacientes enquanto houver condição hemodinâmica e, se possível, os antiarrítmicos (sotalol, amiodarona). Os fármacos com efeito alfa ou beta-adrenérgico – como aminas vasoativas (epinefrina, norepinefrina) e inotrópicos (dobutamina, milrinone) – podem aumentar o risco de arritmias ventriculares; contudo, manter a estabilidade hemodinâmica é soberana em pacientes críticos.

Estima-se que cerca de 17% dos pacientes internados pela Covid-19 necessitam de intubação orotraqueal e ventilação mecânica para recuperação.^1^ A ventilação mecânica tem efeitos hemodinâmicos sobre VD como aumento de pós-carga direita e redução do débito cardíaco direito em pacientes com disfunção do VD e aumento da pressão venosa central.^43^

Os distúrbios eletrolíticos (hipocalemia, hipocalcemia ou hipomagnesemia) podem também aumentar a suscetibilidade em pacientes com substrato anatômico, como é o caso da CAVD e, portanto, a vigilância de eletrólitos deve ser rigorosa.

A hidroxicloroquina e a azitromicina sabidamente prolongam a repolarização ventricular. Dessa maneira, sua associação com os antiarrítmicos da classe III de Vaughan-Williams como sotalol e amiodarona, pode potencializar o risco de atividade deflagrada por pós-potenciais precoces, podendo causar TdP/FV. Conforme sugerido por alguns autores, a amiodarona poderia exercer ação antiviral.^44^ Os antivirais como ritonavir/lopinavir não apresentam efeito catecolaminérgico que aumentem o risco arrítmico em pacientes com cardiopatia e não há evidências que possam interagir com betabloqueadores/antiarrítmicos habitualmente usados na CAVD.^45^

## Taquicardia ventricular polimórfica catecolaminérgica

### Aspectos gerais

A TVPC ocorre em aproximadamente 1 em 10.000 pessoas, acomete principalmente crianças na primeira e segunda década de vida, com síncope ou PCR recuperada, relacionada ao exercício ou à emoção. O ECG de 12 derivações em repouso é normal. O diagnóstico é feito pelo teste de exercício, após a exclusão de doença cardíaca estrutural, preferencialmente por ressonância cardíaca.

**Figura 4:**
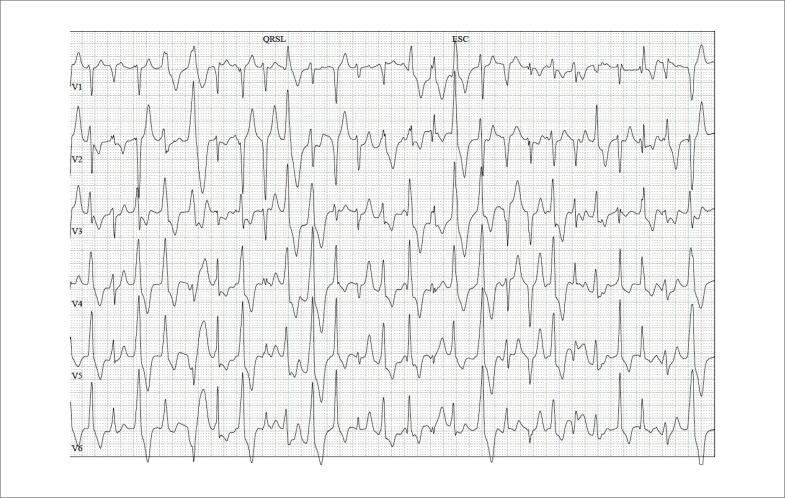
Protótipo da arritmia ventricular em pacientes com taquicardia ventricular polimórfica catecolaminérgica. Paciente de 26 anos de idade, com arritmia ventricular multifocal nas primeiras etapas do esforço físico, com antecedente familiar de morte súbita na primeira década de vida.

Durante o teste de exercício, as extrassístoles ventriculares aparecem com o incremento do esforço físico, quando a FC atinge 100 bpm, progredindo para TV polimórfica e, às vezes, para TV bidirecional clássica que é considerada patognomônica dessa canalopatia (Figura 4).^10^

Fonte: acervo InCor-HC-FMUSP.

A arritmia implicada na TVPC ocorre pela perda de recaptação de cálcio no citosol. A epinefrina, liberada durante o esforço, promove acentuada liberação adicional de cálcio do retículo sarcoplasmático durante a diástole. O acionamento das correntes de sódio e cálcio leva a um acentuado influxo de sódio, que pode despolarizar a célula e provocar extrassístoles por atividade deflagrada por pós-potenciais tardios. A emergência de extrassístoles ventriculares randômicas pelo sistema de Purkinje é responsável pelo aspecto polimórfico da TV.

Aproximadamente 60% dos pacientes com TVPC apresentam defeito no receptor cardíaco de rianodina, codificado pelo gene RyR2 (TVPC tipo 1). A TVPC tipo 2 é mais rara e representa a doença em seu padrão de herança autossômico recessivo, causado por mutações da calsequestrina (CASQ2). Alguns casos de TVPC, ainda mais incomuns, foram relacionados a outras proteínas relacionadas à homeostase do cálcio, gerando o mesmo padrão de arritmias ventriculares. Os genes implicados nessas recentes descobertas foram CALM1 (que codifica a calmodulina) e TRDN (codificação da triadina). As mutações no KCNJ2 e TECRL também já foram descritas.^10^

O objetivo principal da terapia é o bloqueio adrenérgico com propranolol ou nadolol, que pode ser reforçado pela simpatectomia cardíaca esquerda, em pacientes que permanecem sintomáticos ou sem redução das arritmias em testes ergométricos. A flecainida (indisponível no Brasil), recentemente, mostrou benefício no tratamento por inibir a liberação de cálcio mediada pela rianodina (opção, se efeito de classe, com propafenona).^29^

O CDI deve ser indicado principalmente em recuperados de PCR. Entretanto, diferentemente de outras canalopatias, o choque pode induzir a liberação de epinefrina e morte por tempestade elétrica; portanto, a otimização farmacológica é soberana.^46^

### Cuidados na pandemia pela Covid-19

Os pacientes com TVPC, mesmo com controle adequado de sintomas e de arritmias ventriculares, podem apresentar recorrências potencialmente fatais se houver suspensão ou redução dos betabloqueadores e, portanto, é importante manutenção das medicações intra e extra-hospitalares, ponderando o *status* hemodinâmico do doente mais crítico.

Fármacos com efeito alfa ou beta-adrenérgico como aminas vasoativas (epinefrina, norepinefrina) e inotrópicos (dobutamina, milrinone) utilizados habitualmente para suporte hemodinâmico podem aumentar o risco de arritmias ventriculares em pacientes com TVPC. A epinefrina é usada para teste farmacológico na TVPC devido ao seu potencial de desmascarar as arritmias ventriculares, e, portanto, se o paciente necessitar de suporte hemodinâmico, outras aminas vasoativas devem ser preferidas à epinefrina.^7^^,^^47^

O milrinone, inibidor de fosfodiesterase 3, reduz a degradação do monofosfato de adenosina cíclica (AMPc), aumentando a liberação de cálcio pelo receptor de rianodina, que é a patogênese da TVPC. Em algumas situações específicas, considerando o comprometimento hemodinâmico, pode ser possível utilizar dose baixa de bloqueadores de receptores beta 1 (propranolol).^48^

Durante uma infecção grave, os pacientes podem não tolerar os betabloqueadores e antiarrítmicos de uso crônico e, durante todo esse período de maior vulnerabilidade arrítmica, deve-se atentar a distúrbios hidreletrolíticos e evitá-los.

Antivirais como ritonavir/lopinavir não apresentam potencial interação com betabloqueadores ou efeito catecolaminérgico que aumentem o risco arrítmico em pacientes com TVPC, porém podem interagir com a flecainida – fármaco coadjuvante no tratamento da TVPC.^45^ No Brasil, na falta de comercialização de flecainida, utilizamos propafenona, que também pode interagir com os antivirais.

A hidroxicloroquina, aparentemente, não aumenta os níveis de catecolaminas. No entanto, há evidências de interação medicamentosa entre a hidroxicloroquina e o propranolol/nadolol. Os betabloqueadores são metabolizados via citocromo CYP2D6, e sua inibição pela hidroxicloroquina pode acarretar aumento da concentração do medicamento, exigindo monitoramento cuidadoso da frequência cardíaca e da pressão arterial.^49^ A flecainida e a propafenona apresentam uma interação semelhante, resultando em aumento do nível sérico dos antiarrítmicos, potencializando risco arrítmico.^45^ Nessas situações, devemos pesar risco e benefício individual para decisão terapêutica.

## Conclusão

Os pacientes com arritmias hereditárias apresentam diversos fatores moleculares e estruturais que os predispõem a eventos potencialmente fatais no curso de uma infecção viral. A pandemia pela Covid-19 nos leva a distanciá-los do risco de infecção, reforçando medidas de isolamento e higiene, além de orientar precauções nos cuidados médicos, relembrando as peculiaridades de portadores de doenças raras. Dentre as recomendações, reforçamos os cuidados com relação às medicações utilizadas pelo paciente, ao tratamento efetivo da febre e dos distúrbios eletrolíticos e ao risco de prescrição de medicações com potencial pró-arrítmico.
