# Supplementary material for: Peculiaridade dos Pacientes com Arritmias Hereditárias na Pandemia pela COVID-19
Source: Arq Bras Cardiol. 2021 Aug 9;117(2):394–403. [Article in Portuguese] doi: 10.36660/abc.20200391 (PMC8395786; doi:10.36660/abc.20200391)
Supplement: Translation

# Translation: Peculiar Aspects of Patients with Inherited Arrhythmias during the COVID-19 Pandemic

**Authors:** Luciana Sacilotto, Natalia Quintella Sangiorgi Olivetti, Cristiano Faria Pisani, Tan Chen Wu, Ludhmila Abrahão Hajjar, Sissy Lara de Melo, Sávia Christina Pereira Bueno, Esteban Wisnivesky Rocca Rivarola, Muhieddine Omar Chokr, Carina Abigail Hardy, Denise Tessariol Hachul, Francisco Carlos da Costa Darrieux, Mauricio Ibrahim Scanavacca

**Affiliations:** 1 São PauloSP Brazil Instituto do Coração do Hospital das Clínicas da Faculdade de Medicina da Universidade de São Paulo, São Paulo, SP - Brazil.

**Keywords:** COVID-19, Aarrhythmogenic Right Ventricular Cardiomyopathy, Brugada Syndrome, Long QT Syndrome, Catecholaminergic Polymorphic Ventricular Tachycardia

## Abstract

Since December 2019 we have observed the rapid advance of the severe acute respiratory syndrome caused by the new coronavirus (SARS-CoV-2). The impact of the clinical course of a respiratory infection is little known in patients with hereditary arrhythmias, due to the low prevalence of these diseases. Patients who present with infectious conditions may exacerbate hidden or well-controlled primary arrhythmias, due to several factors, such as fever, electrolyte disturbances, drug interactions, adrenergic stress and, eventually, the septic patient’s own myocardial damage. The aim of this review is to highlight the main challenges we may encounter during the Covid 19 pandemic, specifically in patients with hereditary arrhythmias, with emphasis on the congenital long QT syndrome (LQTS), Brugada syndrome (SBr), ventricular tachycardia polymorphic catecholaminergic (CPVT) and arrhythmogenic right ventricular cardiomyopathy.

Since December 2019 we have observed the rapid advance of the severe acute respiratory syndrome caused by the new coronavirus (SARS-CoV-2), the first cases of which arose in Wuhan, China, subsequently arriving in Brazil. Retrospective studies have shown that old age was an independent predictor of mortality by COVID-19. Other risk factors impacting mortality were systemic arterial hypertension, chronic pulmonary obstructive disease, immunosuppression, type-2 diabetes mellitus, obesity, and severe cardiopathy (heart failure, coronary disease, or cardiomyopathies).^1^^,^^2^

Overall, complications due to arrhythmias in patients with pneumonia, particularly atrial fibrillation, are relatively common.^3^^,^^4^ Cardiac arrest occurs in about 3% of hospitalized patients;^5^ however, less than 20% of cardiac rhythms of in-hospital events are reported as being electrically reversible to sinus rhythm (by cardioversion or defibrillation), i.e., ventricular tachycardia/fibrillation (VT/VF).^6^ In such patients, the primary arrhythmogenic mechanism is myocardial injury due to ischemia or inflammation.^4^

The impact of a clinical evolution to sepsis, or specifically of the respiratory infections, is poorly known in patients who harbor inherited arrhythmias, due to the low prevalence of these diseases.^7^ Moreover, most inherited arrhythmias generally have incomplete penetrance and are age-dependent,^8^ being mainly expressed in young patients who, in turn, have a lower risk of developing severe infectious conditions.

Patients presenting with more severe infectious conditions may have exacerbation of concealed or well-controlled arrhythmias, due to several factors such as fever, electrolytic disorders, drug interaction, adrenergic stress and, eventually, the septic patient’s own myocardial injury. All these factors may alter the ion channels balance, rendering these patients with inherited arrhythmias potentially more vulnerable.

Lethal events in patients with inherited arrhythmias may be triggered by physical and emotional stress. The psychosocial impacts of the pandemic, which correlate to depression, stress, anxiety and panic syndrome, further aggravated by social isolation and the confrontation of fear and grief, may all predispose to a higher occurrence of arrhythmias.^9^ The possible need for a temporary suspension of medications (β–;blockers and antiarrhythmic drugs) in hemodynamically unstable patients, the use of vasopressor drugs with catecholaminergic effects, and hydro-electrolytic disorders may be linked to a higher risk for potentially fatal events. Therefore, the pandemic period itself is a warning for the need to warrant vigilance and orientation directed to these patients who, once harboring rare diseases, are not well represented in clinical studies. In case of infection by SARS-COV2, there is not enough epidemiological data on this population to categorize them as a risk group, thus generating insecurity for both physician and patient. In Table 1 we rank the in- and out-of-hospital general healthcare measures that should be adopted for patients with previously known genetic arrhythmias.

**Table 1:** Out-of-hospital and inpatient care during COVID-19 pandemic in patients with inherited arrhythmias

**Out-of-hospital care**
• Avoid emotional stress
• Practice good hygiene and physical distancing
• Check for the risk of social distancing
• Keep beta-blockers and antiarrhythmic drus, as appropriate
• Attention with alarm symptoms (syncope, presyncope and palpitations) and optimal pharmacological treatment and adherence to medication
**Out-of-hospital and inpatient care**
• Keep euthermia – fever should be treated early, especially in patients with LQTS2 and BrS
• Azithromycin and Hydroxychloroquine/Chloroquine must be discouraged in patients with LQTS
• Avoid the association of hydroxychloroquine with amiodarone or sotalol due to the risk of TdP
• The drugs that may lengthen the QT interval is available on www.credibledrugs.org
• Drugs interactions are available on www.online.epocrates.com
• Be aware of QT control protocols of each hospital

LQTS: Long QT syndrome, BrS: Brugada Syndrome, ARVC: arrhythmogenic right ventricle cardiomyopathy; TdP: Torsades de pointes.

For all the aforementioned reasons, it is important to review the main challenges faced during the COVID-19 pandemic,^7^ specifically in this subpopulation, highlighting congenital long-QT syndrome (LQTS), Brugada syndrome (BrS), catecholaminergic polymorphic ventricular tachycardia (CPVT), and arrhythmogenic right ventricular cardiomyopathy (ARVC).

## Congenital Long-QT Syndrome

### Overview

LQTS affects 1:2,000 individuals and is characterized by prolongation of the QT interval on the standard 12-lead ECG and a propensity for syncope or seizures secondary to *torsades de pointes* (TdP) and sudden cardiac death (SDC).^10^

LQTS is associated to defects that either increase the sodium and calcium (respectively Ina and Ica L) depolarization currents, or attenuate the potassium (IKs, IKr and IK1) repolarization currents, leading to a prolonged cardiac action potential that results in QT-interval prolongation.^11^ Clinical peculiarities and molecular diagnosis allow for LQTS classification into subtypes, mainly LQTS-1 (IKs channel, gene *KCNQ1*), LQTS-2 (hERG or IKr channels, gene *KCNH2*), or LQTS-3 (Ina channel, gene *SCN5A*).

The QT interval must be preferably measured from leads DII or V5 (Figure 1) and corrected by the preceding RR interval using Bazett’s formula, ideally at a 60 to 80 bpm heart rate.^12^ The present guidelines define that QTc is prolonged when its value is above 450 ms in men and 460 ms in women. However, 5% to 10% of healthy individuals have a QTc > 460 ms; therefore, other clinical data are necessary to establish LQTS diagnosis. For these cases, the use of Schwartz score is recommended.^13^^,^^14^ There is greater specificity for LQTS only when QTc values are above 480 ms in the absence of secondary causes.^14^ On the other hand, about 30% of the patients have the concealed form of LQTS, represented by genetic background and normal QT interval, so the family history and a genetic test are relevant in case of clinical suspicion in these patients.

**Figure 1:** QT interval measurement in a patient with Long QT Syndrome.

InCor-HCFMUSP collection.

The standard treatment for patients with LQTS is β-adrenergic blockade using nadolol/propranolol, in addition to avoiding drugs that prolong the QT interval. The indication for left cardiac sympathetic denervation and ICD remains restricted to cases with greater risk for potentially fatal arrhythmic events.^14^

### Healthcare during the COVID-19 pandemic

Cardiac ion channels are modulated by the autonomic nervous system, since the cardiac repolarization time is constantly adjusted by the heart rate. In a context of adrenergic stress, there is an increase in heart rate, with potassium channel phosphorylation and increased opening velocity in normal conditions. In situations of genetically-determined protein defect, this adjustment is impaired, thus slowing channel inactivation and allowing for an imbalanced entrance of calcium and occurrence of early after-depolarization (EAD). While the heart rate remains high, there may be some inhibition of these triggers; however, when the metabolic situation leads to bradycardia or RR interval irregularity, there is an increased repolarization dispersion and greater occurrence of EAD. Depending on the cell excitability threshold, EAD gives rise to ventricular extrasystoles (or “premature ventricular beats - PVBs”), TdP/VF.^11^

The suspension or reduction of the chronic use of β – blockers for the treatment of LQTS may aggravate the occurrence of potentially fatal arrhythmias; therefore, there should be a strict maintenance of medications for patients treated at home, while in critically-ill patients, the decision must be guided by their hemodynamic stability.

Ion current balance depends on the cell level of those ions, which can be dynamic in critically-ill patients. Hypokalemia, hypocalcemia and hypomagnesemia lead to the emergence of EAD, increased dispersion of repolarization, and TdP occurs more easily in LQTS carriers. Keeping potassium levels between 4.5 and 5 mg/dL might be a protective strategy.^7^

Sepsis or septic shock are situations associated with high adrenergic tone due to pain/discomfort, in addition to the inflammatory condition itself. LQTS-1 and LQTS-2 carriers are those who present with arrhythmias triggered mainly by adrenergic stress, being therefore more prone to such critical situations. The presence of fever may influence the biophysical properties of temperature-sensitive channels, particularly the hERG channels affected in LQTS-2.^15^ Contrarywise, hypothermia is also associated with prolongation of the QT interval, albeit with a low risk of TdP induction.^16^

Myocardial inflammatory injuries change the cell membrane potential, generating repolarization dispersion and susceptibility to ventricular arrhythmias.^17^ Furthermore, cytokines and antibodies may link to the cardiac ion channels and lead to inflammation-induced channelopathy, presumably with greater severity in LQTS patients.^18^

Potassium-channel (hERG) blocking drugs may further prolong the cardiac repolarization, thus increasing the risk for fatal arrhythmias. The indication of these medications must be strictly weighed, especially in out-of-hospital regimens that lack continuous monitoring. In addition, drugs that lead to P450 3A4 (CYP3A4) cytochrome inhibition may further increase the serum level of drugs that prolong the QT interval.^7^

There is a list of drugs in www.crediblemeds.org that includes chloroquine/hydroxychloroquine and azithromycin. In critically-ill patients, other drugs that pose risk are often administered, such as antiemetic (ondansetron and metoclopramide),^19^ antipsychotic (haloperidol), vasoactive (noradrenaline, dobutamine), analgesic (tramadol), and sedative drugs (etomidate, propofol).^20^

The use of chloroquine and hydroxychloroquine, associated or not to azithromycin, is controversial in COVID-19 patients, of which efficacy has been demonstrated *in vitro*,^21^ but still lacking support from clinical studies. In a recent publication, Mazzanti et al. suggested that a cumulative 2g dose of hydroxychloroquine in 5 days, as it is adopted in 30% of all ongoing studies of hydroxychloroquine (www.clinicaltrials.gov), leads to mild prolongation of the QTc interval in patients with normal baseline QTc (average increase of 20 ms), without increasing the risk for life-threatening arrhythmic complications.^22^ In another series of patients with systemic lupus erythematosus, with mean QTc interval of 443 ± 25.3 ms (373 – 518 ms), QTc interval prolongation occurred in 14.2% of patients using chloroquine.^23^ Considering that LQTS patients already are more prone to TdP-type pro-arrhythmias, the use of chloroquine or hydroxychloroquine, mainly in association with azithromycin, must be discouraged in patients with LQTS.^12^

Polypharmacy is an issue requiring multidisciplinary caution on the part of both physicians and pharmacists; in LQTS patients, this becomes an even more relevant concern, due to the risk of imminent sudden death when they are exposed to such drugs. Specifically in these patients, one must promptly discuss the risks and benefits of each medication.

In case of TdP occurrence degenerating into VF, defibrillation and cardiopulmonary resuscitation are required. TdP usually has a self-limited presentation with spontaneous resolution; however, prevention of TdP recurrence is the most challenging factor. Emergency measures include minimizing pro-arrhythmic medications and suppressing factors that give origin to early after-potentials.^24^

The primary measure aimed to suppress EAD in LQTS is to avoid bradycardia and pause episodes. In patients with acquired LQTS it is possible to try pharmacological measures, such as intravenous isoprenaline; on the other hand, in patients with congenital LQTS, overdriving should only be done by atrial or ventricular stimulation, with a temporary (transcutaneous or transvenous) pacemaker implantation. The administration of 2g followed by continuous infusion (3 to 10mg/min) of magnesium sulphate is an adjunct therapy in both circumstances, aiming to reduce the oscillation amplitude at phase 3 of the membrane action potential. In case of refractoriness, sedation may be necessary to cease the adrenergic stimulus.^25^

## Brugada Syndrome

### Overview

BrS affects about 1 in 5,000 people, with a male gender predominance. The BrS diagnosis is established by the ECG, through the presence of ST-segment elevation >2mm in at least one right precordial lead (V1-V2), in standard (fourth intercostal space, ICS) or superior ICS (second or third ICS), (Figure 2) followed by a negative T-wave (type-1 pattern). The main diagnostic and classification challenge is the fact that this is a dynamic ECG pattern in most patients,^14^ therefore it may be spontaneously documented or obtained after a provocative test using specific drugs (e.g., ajmaline).

**Figure 2:** Placement of precordial leads in a patient with Brugada Syndrome. A) Precordial leads placed at superior intercostal space to increase the detection of Brugada type 1 pattern, as demonstrated in panel B. B) An asymptomatic 26-year-old girl, with a familiar history of sudden cardiac death.

InCor-HCFMUSP collection.

In the absence of secondary causes, the presence of a spontaneous BrS type-1 ECG pattern is sufficient for the diagnosis. In cases of BrS induced by fever or provocative tests, it would be necessary to add personal and familiar clinical data to precisely define the diagnosis. Recently, a scoring system (Shanghai score) was proposed for BrS, which may be used as a diagnostic tool.^26^

Secondary causes, the so-called BrS phenocopies, include the use of drugs that induce the elevation of the ST-segment (e.g. tricyclic drugs), electrolytic disorders, myocardial ischemia, and other forms of ST-segment distortion (presence of *pectus excavatum*) (Table 2).^27^

**Table 2:** Brugada Syndrome phenocopies

**Metabolic conditions**
Adrenal crisis, metabolic acidosis, hyperglycemia
**Electrolyte disturbances**
Hyperkalemia, hypokalemia and hyponatremia
**Mechanical compression of right ventricle outflow tract**
Tumors, pectus excavatum
**Others**
Myocardial ischemia and pulmonary embolism
Pericarditis and myocarditis
Exogenous Medications and Poisonings

Fonte: www.brugadadrugs.org^50^

BrS genetics is more complex than the other primary electrical syndromes. *SCN5A*, the first identified gene, still remains a causative gene; however, all the other 20 genes reported in the recent literature lack a genotype-phenotype correlation.^28^

Therapy for BrS patients involves the avoidance of situations that facilitate the occurrence of potentially fatal arrhythmias (VT/VF), such as fever, use of illicit drugs, alcohol consumption, copious meals, or drugs that increase sodium channel blockade. Quinidine, an important I_to_ channel blocker, seems to be safe and able to reduce arrhythmic events in the clinical follow-up of high-risk patients.

The indication for ICD implantation is restricted to patients that have shown documented spontaneous sustained VT, aborted sudden cardiac death (aSCD), or as primary prevention for those presenting with a greater risk for arrhythmic events, like the presence of syncope. The electrophysiologic study (EPS) can be used for risk stratification of asymptomatic patients, with controversial results.^14^ Radiofrequency ablation of the BrS substrate has emerged as an adjuvant therapy for recurrent ventricular arrhythmias and is being studied in patients without previous arrhythmic events.^29^

### Healthcare in the COVID-19 pandemic

The first aspect to be considered in BrS patients is diagnostic accuracy, since this is a specific electrocardiographic pattern vulnerable to interpretation bias. The list of diseases that can mimic BrS ECG changes, the so-called phenocopies, must be carefully scrutinized for adequate guidance. (Table 2) It is possible to observe that many phenocopies may occur in an infectious condition, such as in myocarditis, electrolytic disorders, pulmonary thromboembolism and myocardial infarction.

Recording ECGs by placing the precordial leads at higher ICS, as depicted in Figure 3, increases the sensitivity for detecting Brugada-type ECG pattern, and may be preferred over the standard lead positioning in patients with suspected or confirmed diagnosis. It can be additionally performed in COVID-19 patients with a VT/VF cardiac arrest during ICU hospitalization, especially if associated with fever. On the other hand, for the analysis of common pathologies during the course of infection, such as myocarditis and infarction, placing precordial leads at higher ICS should be clearly identified in the ECG, to avoid misinterpretation related to R-wave progression, axis and QRS amplitude. QT interval measurement may be performed from D2 of the ECG with superior leads, since the peripheral leads are kept in standard position.

**Figure 3:** ECG of a patient with Arrhythmogenic right ventricle cardiomyopathy. A 45-year-old man recovered from sudden cardiac death. Presence of epsilon wave (arrow) and T wave inversion from V1 to V3, both major signals according to task force criteria. There is also low voltage QRS in limb leads, showing a severe ventricular impairment.

InCor-HCFMUSP collection.

The main measures for the management of BrS patients in the ICU are the prevention of fever and the avoidance of certain medications that enhance channel defects (www.brugadadrugs.org). There are several examples of drugs that increase the risk of sudden death in BrS carriers, such as supportive drugs in hospitalized patients (diphenhydramine), drugs to treat arrhythmias (amiodarone and propafenone), anesthetic agents (propofol), and analgesic drugs (tramadol).

The importance of fever in patients with BrS is well established. Usually, there is an increase of the PR interval, QRS duration, and the J point. Fever also increases the risk of arrhythmia in people older than 70 years in whom the risk of the disease has already been reduced.^30^ Those considered high-risk patients, with temperature over 38.5 ºC even after the administration of antipyretic drugs, should seek medical assistance.^7^

BrS was the first genetic arrhythmia to gain the spotlight in publications during the pandemic. The case of a patient who had the electrocardiographic BrS pattern unmasked by fever was reported. Because this was a young patient to whom the criteria for hospitalization would not apply, the authors chose to discharge him with *LifeVet*, which is not available in Brazil.^31^

Hydroxychloroquine and azithromycin may be indicated, depending on the evaluation of risks *versus* benefits. We suggest measuring serum electrolytes and considering continuous monitoring during the treatment. Prolongation of the QT interval and dispersion of repolarization with hydroxychloroquine and azithromycin might increase the risk for arrhythmia in patients with BrS, even without a direct relationship of these drugs with the depolarization channels.^23^^,^^32^

The management of electrical storm in BrS intends to increase the (depolarization) calcium channel to attain normalization of the ST segment elevation and the reduction of phase 2 reentries. Isoproterenol, as well as β-adrenergic agonists, can be effective, preferably in association with quinidine (unavailable in Brazil). The use of phosphodiesterase III inhibitors, such as oral cilostazol or intravenous milrinone, have shown a decrease in arrhythmogenicity in experimental models of BrS; however, studies on their effects in human beings are still under way.^33^

## Arrhythmogenic Right and/or Left Ventricular Cardiomyopathy

### Overview

The arrhythmogenic right and/or left ventricular cardiomyopathy (AR+/LVC), for a long time known as arrhythmogenic right ventricular dysplasia (ARVD), has an average prevalence of 1:5,000 among the general population.^34^ This heterogeneous disease with various clinical presentations can have sudden cardiac death as its first manifestation, occurring more often during physical exercise. There is no diagnostic standard gold test for AR+/LVC; there is a complex set of clinical tests that comprises the clinical history, electrocardiographic changes of depolarization and repolarization (Figure 3), cardiac imaging, anatomopathological and molecular evaluations.^35^

Currently, AR+/LVC is known to be a genetically-determined, mostly autosomal dominant condition, displaying some rare forms of autosomal recessive inheritance, such as in Naxos Disease^36^ or Carvajal syndrome.^37^ AR+/LVC penetrance is incomplete, the family is affected in up to 50% of the cases, and cases may still be underestimated due to the variant expression of the disease.

AR+/LVC clinical manifestation frequently emerges between the second and fourth decades of life.^38^^,^^39^ The most common symptoms are palpitations, syncope, aSCD and congestive heart failure.

Mutations related to AR+/LVC typically affect genes encoding desmosomal proteins, which are important structures for cardiomyocyte cell adhesion, that play a key role in its physiopathology. Desmosomes, specialized structures of cell connection, are also important mediators acting in the intra- and inter-cell signal transduction.^40^ Total loss of the complex desmosomal function leads to rupture of the cell-cell junction, detachment of myocytes and cell death. Fibro-fatty replacement of the cardiomyocytes contributes to the development of slow conduction areas that generate a scarred anatomic substrate for macro-reentry and ventricular arrhythmias. Fibrosis progresses from the epicardium to the endocardium, involving mainly the right ventricular free wall and causing its aneurysmatic thinning and dilation.^41^

The treatment is focused on the AR+/LVC clinical manifestation. There is no evidence that antiarrhythmic drugs prevent sudden death, and ICD is the indicated management for high-risk patients (aSCD and spontaneous VT). β–blockers are considered to be the first-line therapy for atrial arrhythmias, premature ventricular contractions (PVC), non-sustained VT, besides being important adjuvants for the control of appropriate or inappropriate ICD shocks (especially due to atrial arrhythmias). Sotalol, amiodarone and radiofrequency ablation may be therapeutic alternatives when β–blockers are either ineffective or poorly tolerated.^42^

### Healthcare in the COVID-19 pandemic

Ventricular arrhythmias in AR+/LVC patients are often triggered by physical and emotional stress and have an important adrenergic-dependent component. Thus, the increased adrenergic release related to the compensatory response to the inflammatory syndrome accompanying the infectious condition may induce ventricular arrhythmias. β–blockers should be maintained for as long as the hemodynamic condition persists in these patients and, if possible, also the antiarrhythmic drugs (sotalol, amiodarone). Drugs with alpha- or beta-adrenergic effects, such as vasoactive amines (epinephrine, noradrenaline) and those with inotropic effects (dobutamine, milrinone) may increase the risk for ventricular arrhythmias; however, maintaining the hemodynamic stability is mandatory in critically-ill patients.

It is estimated that about 17% of patients hospitalized with COVID-19 need orotracheal intubation and mechanical ventilation for their recovery.^1^ Mechanical ventilation has hemodynamic effects on the right ventricle, such as the increase of the right afterload and reduction of the right cardiac output in patients with right ventricular dysfunction and increased central venous pressure.^43^

The electrolytic disturbances (hypokalemia, hypocalcemia or hypomagnesemia) can also increase the susceptibility in patients with the anatomic substrate as it is the case in AR+/LVC; therefore, a thorough monitoring of electrolytes must be maintained.

Hydroxychloroquine and azithromycin are known to prolong ventricular repolarization. Thus, their association with class III Vaughan-Williams antiarrhythmics, such as sotalol and amiodarone, may enhance the risk for EAD-triggered activity and TdP/VF. An anti-viral effect of amiodarone has been proposed.^44^ Antivirals such as ritonavir/lopinavir do not have the catecholaminergic effect of increasing the arrhythmic risk, and there is no evidence of drug-to-drug interaction with β–blockers / antiarrhythmic drugs in patients with AR+/LVC.^45^

## Catecholaminergic Polymorphic Ventricular Tachycardia (CPVT)

### Overview

CPVT occurs in approximately 1 in 10.000 people, affecting mainly children in their first and second decades of life, with syncope or aSCD related to exercise or emotion. Resting ECG is within the normal parameters. Diagnosis is attained by performing a stress test, after ruling out cardiac structural disease, preferably by cardiac magnetic resonance imaging.

During the exercise test, premature ventricular beats arise with the increment of physical effort when the heart rate reaches 100 bpm, progressing to polymorphic VT and sometimes to the classic bidirectional VT, which is considered to be pathognomonic for this channelopathy (Figure 4).^10^

**Figure 4:** Ventricular arrhythmia prototype in patients with Cathecolaminergic Polymorphic Ventricular Tachycardia. A 26-year-old girl with polymorphic premature beats and non-sustained ventricular tachycardia during treadmill test and a family history of sudden cardiac death in the first decade of life.

InCor-HCFMUSP collection.

The arrhythmia implicated in CPVT occurs through the loss of calcium reuptake in the cytosol. Epinephrine release during exertion promotes an additional increase in calcium release in the sarcoplasmic reticulum during diastole. The triggering of the sodium and calcium currents leads to a sudden sodium inflow that can depolarize the cell and cause PVCs by delayed afterdepolarization. The emergence of random premature ventricular beats by the Purkinje system is responsible for the polymorphic aspect of the VT.

Approximately 60% of the patients with CPVT have a defective ryanodine cardiac receptor encoded by the RyR2 gene (CPVT type 1). CPVT type 2 is rarer and represents the disease with an autosomal recessive pattern of inheritance, caused by calsequestrin (CASQ2) mutations. Some cases of CPVT, even though unusual, have been related to other proteins associated with the calcium homeostasis, which give origin to the same ventricular arrhythmia pattern. The genes implicated in these recent discoveries are CALM1 (encoding calmodulin) and TRDN (encoding triadin). KCNJ2 and TECRL mutations have been previously described.^10^

The main goal of the therapy is the adrenergic blockade with propranolol or nadolol, which can be reinforced by left cardiac sympathetic denervation in patients who remain symptomatic or with no reduction of PCV and Non-Sustained Ventricular Tachycardia (NSVT) burn during exercise testing. Flecainide, which is not available in Brazil, has been recently shown to have therapeutical benefits by inhibiting ryanodine-mediated calcium release (perhaps propafenone might carry a class effect.^29^

ICD must be indicated mainly in aSCD patients. However, in contrast to other channelopathies, shock may induce release of adrenaline and death by electrical storm and, therefore, pharmacologic optimization is mandatory.^46^

### Healthcare in the COVID-19 pandemic

CPVT patients, even if appropriately controlled for symptoms and ventricular arrhythmias, may present with potentially fatal recurrences if β–blockers are withhold or reduced; therefore, it is important to maintain the in- and out-of-hospital medications for these patients, by assessing the hemodynamic status of critically-ill patients.

Medications with α- or β-adrenergic effects, such as vasoactive (epinephrine, noradrenaline) and inotropic (dobutamine, milrinone) drugs, usually employed for hemodynamic support, may increase the risk for ventricular arrhythmias in CPVT patients. Epinephrine is used as a pharmacological test in CPVT due to its potential for unmasking ventricular arrhythmias, and so, if the patient needs hemodynamic support, other vasoactive amines should be preferred to epinephrine.^7^^,^^47^

Milrinone, a phosphodiesterase-3 inhibitor, reduces cAMP (cyclic adenosine monophosphate) degradation, thus increasing calcium release by the ryanodine receptor, which is the pathogenesis of CPVT. In some specific situations, and considering the hemodynamic compromise, it may be possible to use a low dose of β–1 receptor blocker (propranolol).^48^

In the course of a severe infection, the patients may not tolerate the chronic use of β–blockers and antiarrhythmics and one must pay attention to hydroelectrolytic disorders during the entire period of greater arrhythmic vulnerability, aiming to avoid them.

Antivirals, such as Ritonavir/Lopinavir, do not show any potential interaction with β–blockers nor a catecholaminergic effect that may increase the arrhythmic risk in CPVT patients; however, they may interact with Flecainide – an adjuvant drug in the treatment of CPVT.^45^

Hydroxychloroquine apparently does not increase catecholamine levels. However, there is evidence of drug interaction between hydroxychloroquine and propranolol/nadolol. β–blockers are metabolized through cytochrome CYP2D6, and its inhibition by hydroxychloroquine may result in increased concentration of the drug, which demands careful heart rate and blood pressure monitoring.^49^ Flecainide and propafenone show a similar interaction, resulting in a serum level increase of the antiarrhythmic drugs and enhancing the arrhythmic risk.^45^ In these situations, one must weigh the individual benefit/risk ratio to make a therapeutic decision.

## Conclusion

Patients with inherited arrhythmias present with various molecular and structural factors that predispose them to potentially fatal events in the course of a viral infection. The COVID-19 pandemic prompts us to keep these patients away from the risk of infection and to reinforce measures of isolation and hygiene, in addition to orienting healthcare precautions by recalling the peculiarities of carriers of rare diseases. Among the recommendations, we emphasize caution regarding the medications used by the patient, the effective treatment of fever and electrolytic disturbances, and the risk of prescribing medications with proarrhythmic potential.
